# Vitamin D Serum Levels and the Development of Intensive Care Unit-Acquired Weakness: Insights from a COVID-19 Intensive Care Cohort

**DOI:** 10.3390/pathophysiology32020021

**Published:** 2025-05-09

**Authors:** Jelena Gulišija, Vesna Čapkun, Stefan Golic, Sanda Stojanović Stipić

**Affiliations:** 1 Department of Neurology, University Hospital of Split, 21000 Split, Croatia; 2 Department of Nuclear Medicine, University Hospital of Split, 21000 Split, Croatia; vesna.capkun@gmail.com; 3 School of Medicine, University of Split, 21000 Split, Croatia; sg81938@mefst.hr; 4 Department of Anesthesiology and Intensive Care Unit, University Hospital of Split, 21000 Split, Croatia; sandastojanovicstipic@gmail.com

**Keywords:** ICU-AW, critical illness, COVID-19 pneumonia, vitamin D, respiratory failure, mechanical ventilation

## Abstract

**Background/Objectives**: The pathogenesis of intensive care unit-acquired weakness (ICU-AW) is multi-factorial, with some of the main risk factors being sepsis, multiorgan failure, and the inflammatory response related to critical illness. Vitamin D is crucial for muscle function, the immune response, and inflammation, and has been identified as a predictor of negative outcomes in intensive care unit (ICU) patients with COVID-19. The objective of this preliminary study was to examine the relationship between vitamin D serum levels and the incidence of ICU-AW in a cohort from the University Hospital of Split. **Methods**: A prospective observational cohort study was conducted in the University Hospital of Split in ICU from December 2021 to March 2022. The inclusion criteria were as follows: patients over 18 years old who had a confirmed severe acute respiratory coronavirus disease 2 (SARS-CoV-2) infection, patients who were mechanically ventilated for more than 48 h, and patients who were weaned from a ventilator over at least 24 h. The exclusion criteria were a history of neurological or musculoskeletal disorders and a pre-existing poor functional status. Vitamin D was detected in the first routine blood sample. **Results**: A total of 77 patients were observed, with 36 patients who were successfully weaned from a ventilator over at least 24 h and 1 patient who could not be examined because of impaired consciousness (this patient was excluded from further analysis), and thus a total of 35 patients were analyzed. Of these 35 patients, 12 (34%) developed ICU-AW. The median vitamin D serum level in the ICU-AW group was 17 (7.5–73.3), while that in the non-ICU-AW group was 25.2 (12.3–121). The difference in vitamin D serum levels between the groups was not significantly different from zero (*p* = 0.567). All patients, except for one, were vitamin D insufficient. **Conclusions**: Vitamin D serum levels in the ICU-AW group were not statistically different from the non-ICU-AW group, possibly due to the small sample size. Given the known roles of vitamin D in muscle function, immune modulation, and inflammation, a potential etiopathogenetic role in ICU-AW cannot be excluded without additional studies. Therefore, further studies with larger sample sizes than ours are necessary to determine whether vitamin D deficiency contributes to the development of ICU-AW and whether supplementation could have preventive or therapeutic value.

## 1. Introduction

Infection with severe acute respiratory syndrome coronavirus 2 (SARS-CoV-2) can result in hypoxemic respiratory failure and can require treatment in an intensive care unit (ICU) [1,2,3]. About 50–80% of critically ill patients treated in an ICU develop intensive care unit-acquired weakness (ICU-AW) [4,5,6]. ICU-AW is a diffused generalized muscle weakness that can only be explained by critical illness [7]. This condition can result from critical illness myopathy (CIM), critical illness polyneuropathy (CIP), or a combination of the two, known as critical illness neuromyopathy (CINM) [7,8]. The pathogenesis of ICU-AW is multi-factorial, with the main risk factors being sepsis, multiorgan failure, inflammatory response related to critical illness, mechanical ventilation (MV), hyperglycemia, and exposure to glucocorticoids and neuromuscular blocking agents (NMBAs) [9,10,11]. Older age and, in some studies, female sex are also recognized as risk factors for ICU-AW development, although the specific age threshold varies across studies, with some identifying 59 years and others identifying 62 years as the cutoff [12,13,14,15,16,17]. ICU-AW diagnosis is primarily clinical, backed by muscle strength testing (Medical Research Council test, MRC test) and possibly electrophysiological studies [15]. A vigilant approach and thorough clinical assessment are imperative for the timely recognition and effective management of this condition.

In addition, ICU-AW is associated with unsuccessful weaning from an MV, causing further health complications, reintubation, prolonged hospitalization, and higher mortality. Almost 50% of patients who develop ICU-AW after weaning from an MV will require reintubation, and 50% of them will die during hospitalization [18]. Considering this, effectively and promptly preventing, diagnosing, and treating ICU-AW is crucial.

Vitamin D is crucial for muscle function, immune response, and inflammation, which are critical factors in developing and recovering from ICU-AW [19]. Vitamin D levels impact muscle mass, strength, contraction speed, and the innate and adaptive immune response [20,21]. A deficiency in vitamin D may impair the body’s ability to fight infection and reduce inflammation, both of which are vital for critically ill patients [22]. High levels of systemic inflammation are expected in ICU patients and contribute to muscle degradation and weakness [23]. Vitamin D deficiency is notably associated with increased inflammatory markers [24,25,26,27]. In addition, vitamin D serum levels below 9.9 ng/mL (24.71 nmol/L) at admission are associated with a higher risk of in-hospital mortality in patients with COVID-19 disease [28]. Therefore, we must understand the effects of vitamin D serum levels on the development of ICU-AW in patients with COVID-19 post-ventilation.

Hypovitaminosis D refers to a vitamin D serum level below 75 nmol/L, and it is defined as vitamin D insufficiency (vitamin D levels below 75 nmol/L), vitamin D deficiency (vitamin D levels below 50 nmol/L), and vitamin D severe deficiency (vitamin D levels below 30 nmol/L) [29]. Vitamin D serum levels below 30 nmol/L are associated with osteomalacia, myopathy, and insufficient reabsorption from the intestine [30,31]. In comparison, levels from 50 nmol/L to 75 nmol/L are associated with osteoporosis and increased PTH values [30,31]. The recommended vitamin D serum level is more than 75 nmol/L for the skeletal system and more than 120 to 130 nmol/L for anticancer effects, while the optimal blood level for the muscle system is yet to be established [29].

Despite its crucial role in several health functions, vitamin D is deficient across all age groups and populations worldwide [32]. Roughly 50% of the world’s population and up to 80% of critically ill patients in ICUs are vitamin D deficient [33,34,35,36]. Groups at risk of developing a vitamin D deficiency are pregnant women, nursing mothers, breastfed infants, individuals with limited or poor-quality sun exposure, individuals with obesity, children under five years old, people with dark skin pigmentation, individuals aged 65 or above, individuals with malabsorption syndrome, and individuals with kidney disease [30,31]. Postmenopausal women appear to be at a higher risk of vitamin D deficiency due to several factors. These include hormonal fluctuations, the use of excessive clothing or sunscreen that limits sun exposure, changes in body fat composition, diets lacking in vitamin D, and a sedentary lifestyle; however, there is also a notable reduction in the skin and kidneys’ capacity to produce vitamin D and a decrease in intestinal absorption [37,38,39].

Observational studies indicate an association between low vitamin D levels and muscle weakness in children and older people [40,41,42]. Children with a hereditary deficiency of active vitamin D (i.e., a genetic deficiency of CYP27B1) have severe muscle weakness, which is rapidly improved by treatment with the activated form of vitamin D [43]. A meta-analysis of 29 trials showed a small but significant improvement in global muscle strength with vitamin D supplementation, although this meta-analysis was limited by substantial heterogeneity [44]. In another meta-analysis, among four studies in patients with baseline vitamin D concentrations less than 25 nmol/L, three showed improved muscle strength with vitamin D supplementation [45]. The combination of vitamin D supplementation and strength exercises [46], or the addition of vitamin D to protein supplementation, was demonstrated to enhance muscle strength in individuals with sarcopenia [47,48]. However, vitamin D supplementation alone did not yield the same effect in this population [49]. The most effective strategy for vitamin D supplementation in preventing and treating sarcopenia involves the use of vitamin D supplements alongside sufficient protein intake and regular exercise [50,51]. Considering the evidence of vitamin D levels’ effect on muscle weakness and overall health, vitamin D could play a role in the development of ICU-AW, while vitamin D supplementation in deficient patients could prevent ICU-AW. However, the association between ICU-AW and vitamin D has not been examined.

The objective of this preliminary study was to examine the relationship between vitamin D serum levels at ICU admission and ICU-AW incidence in ICU settings using data on 35 patients at the University Hospital Split in the winter of 2021–2022.

## 2. Materials and Methods

This prospective observational cohort study was conducted in the COVID ICU at the University Hospital of Split in Croatia from December 2021 to March 2022 and involved 77 patients, of whom 35 met the inclusion criteria and were analyzed, comprising 23 male and 12 female subjects. All patients were ethnically Croatian. The inclusion criteria were as follows: patients over 18 years old with a SARS-CoV-2 infection confirmed through a real-time reverse transcription polymerase chain reaction test. The subjects were admitted to the ICU due to respiratory insufficiency caused by COVID-19 pneumonia, mechanically ventilated for more than 48 h, and weaned from a ventilator over at least 24 h. The exclusion criteria were a history of neurological or musculoskeletal disorders and a pre-existing poor functional status (modified Rankin score ≥ 4) [52]. The patients were asked for consent in the presence of a witness (one of the medical staff), who later signed an informed consent form in the non-infectious area.

The following clinical characteristics were collected from the subjects: age, sex, Sequential Organ Failure Assessment (SOFA score), Acute Physiology and Chronic Health Evaluation II (APACHE score II), comorbidities, vaccination status, and the number of days from COVID-19 disease to respiratory failure.

The patients were examined the day after analgosedation was abolished and after weaning from the MV. To participate in this study, the patients were required to be awake, oriented, and cooperative at the time of examination and to score 15 points on the Glasgow Coma Scale.

The primary outcome variable was muscle strength, as objectified using the MRC test during the morning from 8 to 12 a.m. A value of 48–60 indicated satisfactory muscle strength (the non-ICU-AW group), and a value less than 48 showed the development of ICU-AW (the ICU-AW group).

The secondary outcome variables were the vitamin D serum level on the first day after ICU admission, the need for reintubation, the need for tracheotomy, the number of days of MV, the length of the ICU stay, and the duration of hospitalization.

Vitamin D (25-OH vitamin D serum level) was detected in the first routine blood sample, usually taken on the first morning in the ICU, the day after admission, and it was measured once. Blood was collected in vacutainer tubes and measured in the hospital laboratory using the ECLIA method (Elecsys Vitamin D total III kit; Roche Diagnostics, Diegem, Belgium). Patients who were vitamin D insufficient (<75 nmol/L) received vitamin D—4000 IU of cholecalciferol daily (1 mL of Plivit D_3_ © 4000 IU oral suspension 4000 IU/mL, Pliva, Zagreb, Croatia). The supplement was started on the first morning after ICU admission and administered orally or via a gastric tube during the ICU stay.

The data were processed with the statistical package IBM SPSS 20. Qualitative variables were presented as absolute and relative numbers, and quantitative variables were presented as medians (min–max). The distributions of qualitative variables between the examined groups were compared using Fisher’s exact test. The Mann–Whitney U test was used to compare quantitative data between the two groups. The results were interpreted at a significance level of 95%. This study is an exploratory pilot study, so no formal power calculation was performed.

This study was conducted in accordance with the Declaration of Helsinki of the World Medical Association for experiments involving humans. The institutional ethics committee of the University Hospital of Split approved the study protocol (No. 500-03/22-01/37).

## 3. Results

During the period from December 2021 to March 2022, a total of 77 patients were observed in the COVID-19 ICU at the University Hospital of Split [53]. In this group, 36 (47%) patients were successfully weaned from a ventilator over at least 24 h. One patient could not be examined because of impaired consciousness (this patient was excluded from further analysis). A total of 35 patients were analyzed. A study flow diagram is presented in Appendix A. The characteristics of these patients are presented in Table 1.

A previous study conducted on this cohort [53] examined and found that, out of the 35 patients, 12 (34%) developed ICU-AW. The median age in the ICU-AW group was 71 years (58–77), and in the non-ICU-AW group was 66 years (47–75), which was statistically different (*p* = 0.038). In the ICU-AW group, 58% of the patients were women, and, in the non-ICU-AW group, 78% of the patients were men, with no statistical difference. In the ICU-AW group, five patients (42%) died during hospitalization, whereas in the non-ICU-AW group, only one patient (4%) died (*p* = 0.012) [53]. Other results of the previous study are presented in Table 2 [53].

Here, in contrast, we analyzed the differences in vaccination status, number of days from SARS-CoV-2 infection to respiratory failure, comorbidities, SOFA score, and APACHE II score between ICU-AW and non-ICU-AW groups, along with focusing on vitamin D serum levels. In the ICU-AW group, 11 out of 12 (92%) patients were not vaccinated, and, in the non-ICU-AW group, 19 out of 23 (82%) patients were not vaccinated, with no statistical significance (*p* = 0.640). The number of days from SARS-CoV-2 infection to respiratory failure in the non-ICU-AW group was 9 (2–15), and in the ICU-AW group was 7.5 (6–17), with no statistical difference (*p* = 0.888). There was no statistical significance in comorbidities between the groups (*p* = 0.113). The APACHE II score at ICU admission was 10 (5–19) in the ICU-AW group and 9 (3–16) in the non-ICU-AW group, with no statistical significance (*p* = 0.151). The SOFA score at ICU admission was 2 (2–7) in the ICU-AW group and 2 (2–3) in the non-ICU-AW group, with no statistical significance (*p* = 0.144). The observed median vitamin D serum level in the ICU-AW group was 17 (7.5–73.3), while that in the non-ICU-AW group was 25.2 (12.3–121), with no statistically significant difference (*p* = 0.567). All patients, except for one, were vitamin D deficient (Table 3). Notably, 29 (82%) patients were vitamin D deficient (below 50 nmol/L), of whom 20 (57%) patients were severely vitamin D deficient (below 30 nmol/L), 5 (14%) patients were vitamin D insufficient (50–75 nmol/L), and only 1 (3%) patient was vitamin D sufficient (Table 3). We also compared the incidence of ICU-AW in the group of patients whose vitamin D serum level was below 30 nmol/L with that in the group of patients whose vitamin D serum level was above 30 nmol/L, and there was no statistically significant difference (*p* = 0.488, Table 4).

## 4. Discussion

This study found no statistically significant difference in vitamin D serum levels between the ICU-AW and non-ICU-AW groups. Although our results did not reveal a statistically significant association between vitamin D insufficiency and ICU-AW, the potential role of vitamin D in muscle physiology, immune modulation, and recovery remains biologically plausible [19,20,21,22,23,24,25,26,27]. Given the presence of systemic inflammation and prolonged immobilization typical of ICU-AW [23], vitamin D deficiency may contribute to the exacerbation of muscle weakness. Further research with a larger sample size is required to clarify whether the association exists. If confirmed, this could open the possibility for vitamin D supplementation to serve as a potential strategy for the prevention and treatment of ICU-AW. Previous research suggested that a vitamin D level below 9.9 ng/mL (≤24.71 nmol/L) at admission is associated with increased in-hospital mortality in patients with COVID-19 [28]. In our ICU-AW group, the median vitamin D level was below this threshold (17 nmol/L), which may be clinically relevant. Although the difference in vitamin D levels between the ICU-AW and non-ICU-AW groups was not statistically significant, the fact that the ICU-AW group had median vitamin D levels below the mortality-associated cut-off indicates that the ICU-AW group may still be considered at higher risk based on previous results. In addition, it is important to emphasize that, in our study, only 1 patient (3%) was vitamin D sufficient, while 34 patients (97%) were vitamin D insufficient and 20 patients (57%) were severely vitamin D deficient (Table 3). In a study by Bychinin et al., all 40 patients with COVID-19 admitted to ICU had low vitamin D levels [12 (9–15) ng/mL (29.95 (22.46–37.44) nmol/L)] [28]. Therefore, detecting vitamin D levels in critically ill patients needs to be a standard in ICU care.

Increased age was detected as a significant risk factor for ICU-AW development in this cohort (*p* = 0.038) [53], consistent with previous research [12,13,14]. The median age in the ICU-AW group was 71 years, which was 5 years higher than that in the non-ICU-AW group, further supporting the association between aging and increased susceptibility to ICU-AW. Additionally, an age older than 65 years is a known risk factor for vitamin D deficiency [30,31,40,41,42]. Sex differences were also observed. In this cohort, males were predominant in the non-ICU-AW group (78% of the patients in the non-ICU-AW group were men), while females were predominant in the ICU-AW group (58% of the patients who developed ICU-AW were women). However, this difference did not reach statistical significance (*p* = 0.059) [53]. The findings of this cohort do not align with previous studies, which identified female sex as a risk factor for ICU-AW development [15,16,17]. This discrepancy may be attributed to the small sample size of this cohort. Additionally, the association between female sex and ICU-AW risk could be linked to the fact that postmenopausal women are more susceptible to vitamin D deficiency [37,38,39]. Due to the limited sample size, we could not analyze all contributing factors in the same model, particularly vitamin D levels. This limitation should be considered when interpreting the results.

When examining vitamin D levels in relation to insufficiency, deficiency, severe deficiency, and the development of ICU-AW (Table 3), a difference in vitamin D levels was observed between ICU-AW and non-ICU-AW groups. Considering that a vitamin D level below 30 nmol/L is associated with underlying myopathy [29], and that the observations per group for levels above 30 nmol/L were sparse, a vitamin D level of 30 nmol/L was set as a threshold for ICU-AW development and all observations for levels above 30 nmol/L were collapsed into a single category (Table 4). However, the incidence of ICU-AW in the group of patients whose vitamin D serum level was below 30 nmol/L was not significantly greater (*p* = 0.488, Table 4) than in the group whose vitamin D serum level was above 30 nmol/L. This could be explained by the fact that the ICU-AW diagnosis was made clinically, so it encompassed CIM and CIP, even though a study by Moonen et al. indirectly indicated that myopathy was predominant [54,55]. Although inflammatory response related to critical illness plays a central role in ICU-AW development and myopathy is simply considered an epiphenomenon of the inflammatory response to the viral infection, muscle involvement in COVID-19 is not uncommon, and it can sometimes be challenging to differentiate myopathy from myositis syndrome [56,57,58,59,60,61]. Importantly, SARS-CoV-2 was not detected in muscle immunohistochemistry or electron microscopy, arguing against direct viral myocyte invasion [55]. Therefore, the most likely mechanism for muscle injury in SARS-CoV-2 is the virus-triggered activation of innate and adaptive immunity [55]. Considering polyneuropathy and CIP, all variants of Guillain Barre syndrome are associated with unknown prevalence [62,63,64,65]. Given our limited sample size, we encourage future research to expand upon these findings with larger cohorts to better understand the potential role of vitamin D in ICU-AW development.

In some previous studies, vitamin D supplementation was shown to have benefits regarding muscle strength and contractility [43,44,45]. However, in other studies, vitamin D as a monotherapy did not show this effect [50,51]. The most significant benefit of vitamin D supplementation is likely to occur in patients with baseline vitamin D concentrations below 25 nmol/L [66]. However, dosage and dosing protocols have yet to be clearly defined. In our study, all patients who were vitamin D insufficient were given vitamin D orally at a daily dose of 4000 IU. A systemic review conducted by Pal et al. [67] indicated that vitamin D supplementation is associated with improved clinical outcomes in patients affected by COVID-19. Most analyzed studies used oral cholecalciferol, with cumulative doses ranging from 80,000 to 400,000 IU [67]. When defining the dose regimen, it is essential to consider that vitamin D functions as an anabolic hormone during periods of physiological catabolism as part of a systemic inflammatory response [19,20,21,22,23].

In this study, 12 (34%) patients developed ICU-AW [53]. In a study by Schmidt et al., the incidence of ICU-AW was 52% at the time of weaning from MV [68]. Van Aerde found that the incidence of ICU-AW in patients with COVID-19 at the time of weaning from MV was 72% [5]. This difference could be explained by the fact that the study by Schmidt et al. included participants who used noninvasive ventilation and a high-flow nasal cannula; when analyzing only the participants who needed MV, the incidence was 52% at the time of weaning from MV [68]. Still, we assume that our study’s total number of patients who developed ICU-AW was higher because we only observed patients on the first attempt to wean from MV. Five patients (22%) in the group that returned to MV were not monitored for weaning attempts [53].

In this cohort, almost half of the patients (*n* = 5) in the ICU-AW group died, while, in the non-ICU-AW group, only one patient (4%) (*p* = 0.012) died [53], which roughly corresponds to the mortality observed during hospitalization in other studies [18,69,70]. Research conducted by Ali et al. found lower mortality during hospitalization in the ICU-AW group (31.4%) [71].

We did not find a statistical difference in the APACHE II score (*p* = 0.151) or SOFA score (*p* = 0.144) at the time of ICU admission between the ICU-AW and non-ICU-AW groups. However, Feng et al. found significant differences in the APACHE II and SOFA scores on the first day of ICU admission between the ICU-AW and non-ICU-AW groups [72]. The small sample size in our study could explain the lack of statistically significant differences in the APACHE II and SOFA scores between the ICU-AW and non-ICU-AW groups. In the study by Feng et al., the APACHE II and SOFA scores were higher, which could be explained by the fact that, in their study, the inclusion criteria were patients who were mechanically ventilated for more than 7 days. In our study, 14 patients (40%) were mechanically ventilated for less than 7 days; thus, it is possible that our study included patients with less severe disease.

Drury et al. showed that vaccination decreased the inflammatory response in COVID-19 disease [73], possibly providing protection against ICU-AW development. In our study, there was a higher proportion of non-vaccinated patients in the ICU-AW group (92%) than in the non-ICU-AW group (83%). However, the small sample size limited the ability to draw conclusions. We did not observe a statistically significant difference (*p* = 0.640) in vaccination status between the groups. Future studies with larger sample sizes may provide more clarity on this potential relationship.

The main limitation of our study was the small number of patients; thus, we consider this a preliminary study. We were not able to include more patients due to the end of the pandemic. Therefore, the results of this study should be interpreted cautiously, and no causal conclusion can be drawn. This study could be included in more extensive studies. Body mass was not measured, and therapy that could also affect ICU-AW development (analgosedation, NMBA, corticosteroid therapy, and antibiotics) was not analyzed. In this study, the diagnosis of ICU-AW was made clinically, and electrophysiological investigations were not performed. Electrophysiological signs of ICU-AW have been found within three days of ICU admission [74], and early detected electrophysiological alterations can be rapidly reversed [75]. It is likely that more patients developed ICU-AW, and, by the time they were weaned from MV, their muscle strength returned to normal. In that case, we could detect the critical vitamin D level under which ICU-AW develops.

## 5. Conclusions

In this preliminary study, even though observed vitamin D serum levels in the ICU-AW group were lower compared with vitamin D serum levels in the non-ICU-AW group, there was no statistically significant difference. This result indicates that vitamin D deficiency alone does not play a significant role in ICU-AW development when it comes to critical illness. However, the results should be interpreted cautiously due to the small sample size in combination with the reduced range of vitamin D levels across the groups. It is important to emphasize that all patients, except for one, were vitamin D insufficient and required supplementation, suggesting that detecting vitamin D serum levels should be a standard of ICU care. Further studies are needed to determine whether there is a causal relationship between vitamin D levels and ICU-AW, and the potential benefits of vitamin D supplementation in preventing or treating ICU-AW.

## Figures and Tables

**Table 1:** Characteristics of the cohort of analyzed subjects at ICU admission and comparison of the ICU-AW group with the non-ICU-AW group.

Characteristics	Categories	Total	Non-ICU-AW Group	ICU-AW Group	*p*-Value
Sex	Male	23	18 (78%)	5 (42%)	0.059 *
	Female	12	5 (22%)	7 (58%)	
Age (years)		68 (48–77)	66 (47–75)	71 (58–77)	0.038 †
Vaccinated	Yes	5	4 (17%)	1 (8%)	0.640 *
	No	30	19 (83%)	11 (92%)	
Number of days from SARS-CoV-2 infection to respiratory failure		8 (2–17)	9 (2–15)	7.5 (6–17)	0.888 †
Comorbidities	Hypertension	21	12	9	0.282 *
	Diabetes mellitus	12	5	7	0.059 *
	Cardiovascular disease	3	1	2	0.266 *
	Cerebrovascular disease	2	0	2	0.111 *
	Chronic lung disease	3	3	0	0.536 *
	Malignant disease	3	3	0	0.536 *
	Autoimmune disease	0	0	0	1.000 *
Number of comorbidities	No comorbidities	10	9	1	0.113 *
	1	14	5	5	0.258 *
	2	12	8	4	1.000 *
	≥3	3	1	2	0.266 *
APACHE II score		9 (3–19)	9 (3–16)	10 (5–19)	0.151 †
SOFA score		2 (2–7)	2 (2–3)	2 (2–7)	0.144 †
Vitamin D level		25.3 (7.5–121)	25.2 (12.3–121)	17 (7.5–73.3)	0.567 †

Data are presented as numbers (percentages) or medians (min–max). * Fisher’s exact test; † Mann–Whitney U test.

**Table 2:** Results of statistical analyses of differences between ICU-AW and non-ICU-AW groups.

Outcomes	Description	Total	Non-ICU-AW Group	ICU-AW Group	*p*-Value
Duration of mechanical ventilation (days)	Until first weaning from mechanical ventilation	8 (3–84)	6 (3–54)	13.5 (8–84)	<0.001 †
Weaning from ventilator (number of tries)	1	21 (60%)	18 (78%)	3 (25%)	0.004 *
	≥2	14 (40%)	5 (22%)	9 (75%)	
Duration of mechanical ventilation (days)	Total time	11.5 (4–94)	7 (4–54)	32 (8–94)	0.007 †
Duration of mechanical ventilation less than 7 days in non-ICU-AW group and ICU-AW group		14 (40%)	14 (61%)	0 (0%)	NA
Tracheotomy	Yes	15 (43%)	6 (26%)	9 (75%)	0.011 *
	No	20 (57%)	17 (74%)	3 (25%)	
Number of days in ICU (*n*)		32.5 (8–98)	20 (8–80)	54 (14–98)	0.008 †
Number of days in ward		18 (6–103)	12 (6–81)	45 (10–103)	0.001 †
Final outcome	Discharged home	21 (60%)	17 (74%)	4 (33%)	0.012 *
	Discharged home with oxygenator	8 (23%)	5 (22%)	3 (25%)	
	Mortal outcome during hospitalization	6 (17%)	1 (4%)	5 (42%)	

Data are presented as numbers (percentages) or medians (min–max). * Fisher’s exact test; † Mann–Whitney U test. Source: [53].

**Table 3:** The range of vitamin D serum levels and ICU-AW incidence.

ICUAW		Vitamin D Level		
	<30 nmol/L	30–50 nmol/L	50–75 nmol/L	>75 nmol/L
Yes	8	1	3	0
No	12	8	2	1

Data are presented as numbers.

**Table 4:** Comparison of vitamin D deficits in ICU-AW and non-ICU-AW groups.

	Vitamin D Level		*p*-Value
	<30 nmol/L	>30 nmol/L	0.488 *
ICU-AW group	8	4	
Non-ICU-AW group	12	11	

Data are presented as numbers. * Fisher’s exact test.

## Data Availability

Data are available on request.

## Appendix A

Study flow diagram.
